# FolR1: a novel cell surface marker for isolating midbrain dopamine neural progenitors and nascent dopamine neurons

**DOI:** 10.1038/srep32488

**Published:** 2016-09-01

**Authors:** Nicole Gennet, Claudia Tamburini, Xinsheng Nan, Meng Li

**Affiliations:** 1 Neuroscience and Mental Health Research Institute, School of Medicine and school of Bioscience, Cardiff University, Cardiff, CF24, 4HQ, UK

## Abstract

Cell type-specific surface markers offer a powerful tool for purifying defined cell types for restorative therapies and drug screenings. Midbrain dopaminergic neurons (mesDA) are the nerve cells preferentially lost in the brains of Parkinson’s disease patients. Clinical trials of transplantation of fetal neural precursors suggest that cell therapy may offer a cure for this devastating neurological disease. Many lines of preclinical studies demonstrate that neural progenitors committed to dopaminergic fate survive and integrate better than postmitotic DA neurons. We show that the folate-receptor 1 (FolR1), a GPI-anchored cell surface molecule, specifically marks mesDA neural progenitors and immature mesDA neurons. FolR1 expression superimposes with Lmx1a, a bona-fide mesDA lineage marker, during the active phase of mesDA neurogenesis from E9.5 to E14.5 during mouse development, as well as in ESC-derived mesDA lineage. FolR1^+^ neural progenitors can be isolated by FACS or magnetic sorting (MAC) which give rise to dopamine neurons expressing TH and Pitx3, whilst FolR1 negative cells generate non-dopaminergic neurons and glia cells. This study identifies FolR1 as a new cell surface marker selectively expressed in mesDA progenitors *in vivo* and *in vitro* and that can be used to enrich *in vitro* differentiated TH neurons.

Dopaminergic neurons derived from the ventral mesencephalon (mesDA) are the cells preferentially lost in the brains of Parkinson’s disease patients. Proof of principle has been provided that pluripotent stem cell (PSC)-derived mesDA neural progenitors are able to survive and differentiate into mature dopamine neurons in animal models of Parkinson’s disease and exhibit some functional characteristics^1^,^2^, hence promising hope for the development of cell transplantation therapy for treating Parkinson’s disease. Moreover, there are growing interests in using human neurons derived from patient induced pluripotent stem cells (iPSCs) for understanding the aetiology of Parkinson’s disease and other neurological disorders where mesDA neurons are implicated^3^,^4^,^5^,^6^.

However, notwithstanding the significant development of new robust small molecule based mesDA differentiation protocols^1^,^7^,^8^, PSC-derived dopaminergic cultures still contain other cellular identities such as non-DA neuronal subtypes, non-neural cells and undifferentiated intermediates. From cell therapy standpoint, these ‘undesirable’ cells greatly compromise efficacy and poise risk of tumour formation. One possible measure to guard safety is to transplant a committed dopaminergic cell population with defined molecular characteristics, that can be isolated by fluorescent activated cell sorter (FACS) or magnetic beads using a panel of cell surface markers^9^. Our ability to isolate a defined mesDA cell population will also benefit the field of iPSC-based disease modelling. It is generally acknowledged that different iPSC lines, derived from distinct individuals or even from the same pool of somatic cells, can differ significantly in lineage differentiation potential and other cellular behaviours^3^. Such intrinsic differences invariably increase the noise of the iPSC cellular model system, which in turn either mask subtle cellular phenotypes or lead to false phenotypes.

A number of cell surface markers have been identified to be expressed by mesDA neurons or their progenitors^10^,^11^,^12^. Of these, Corin has been validated as a FACSable epitope for enriching mesDA transplantable progenitors from rodent embryos and hPSC derivatives^13^,^14^. However, Corin expression is also found in non-dopaminergic hindbrain and spinal cord floor plate. Thus, a second marker is required in order to restrict the isolation of PSC-derived neurons to mesDA lineage, for example, the use of an Otx2-GFP reporter mouse ESC line by Chung *et al*.^15^. However, generating reporter cell lines by genetic engineering is not a practical solution for applications that concern multiple lines of reference or patient iPSCs.

Here we report the identification of folate receptor alpha (FolR1) as a highly specific cell surface marker for ventral mesencephalic floor plate neural progenitors and nascent mesDA neurons. We show that cell sorting using FolR1 antibody is able to purify mesDA progenitors, which subsequently give rise to highly enriched cultures of mesDA neurons. We anticipate that the FolR1-based purification of mesDA progenitors would serves as a useful tool for developing Parkinson’s cell therapy and elucidating disease aetiology using patient iPSC lines.

## Results

### Transcription profiling identified FolR1 as a candidate mesDA surface marker

We have previously carried out a RNA microarray profiling to identify genes preferentially expressed in the developing midbrain floor plate that give rise to mesDA neurons^16^. This screen resulted in a list of 87 candidate floor plate/mesDA progenitor marker genes encoding cell membrane proteins (Fig. S1, supplementary Table 1). We performed an *in silico* gene expression screening of these candidates using publically available databases such as the Eurexpress and Allen Brain Atlas *in situ* hybridisation database (http://www.eurexpress.org/ee/; http://www.brain-map.org/). Expression of 65 genes was found in a database, of which 45 showed ventral midbrain expression (supplementary Table 1). This short list include the previously reported floor plate cell surface molecule Corin and Alcam, which are expressed in, but not restricted to, the ventral midbrain^10^,^17^. We then carried out a pilot immunohistochemical analysis of 7 candidates for which with best expression patterns and that an antibody is commercially available. These candidates include Folate receptor alpha (FolR1), Annexin A1(Anxa1), Annexin A2 (Anxa2), Growth hormone receptor (GHR), G protein-coupled receptor 37 (Gpr37), Cadherin 6 (Cdh6) and plexin domain containing 2 (Plxdc2). From the expression study of E10.5 mouse embryos, FolR1 showed the most promising expression pattern in the ventral midbrain and therefore we focused the studies on this marker subsequently.

### FolR1 expression marks mesencephalic dopaminergic neurogenic zone

**Figure 1:**
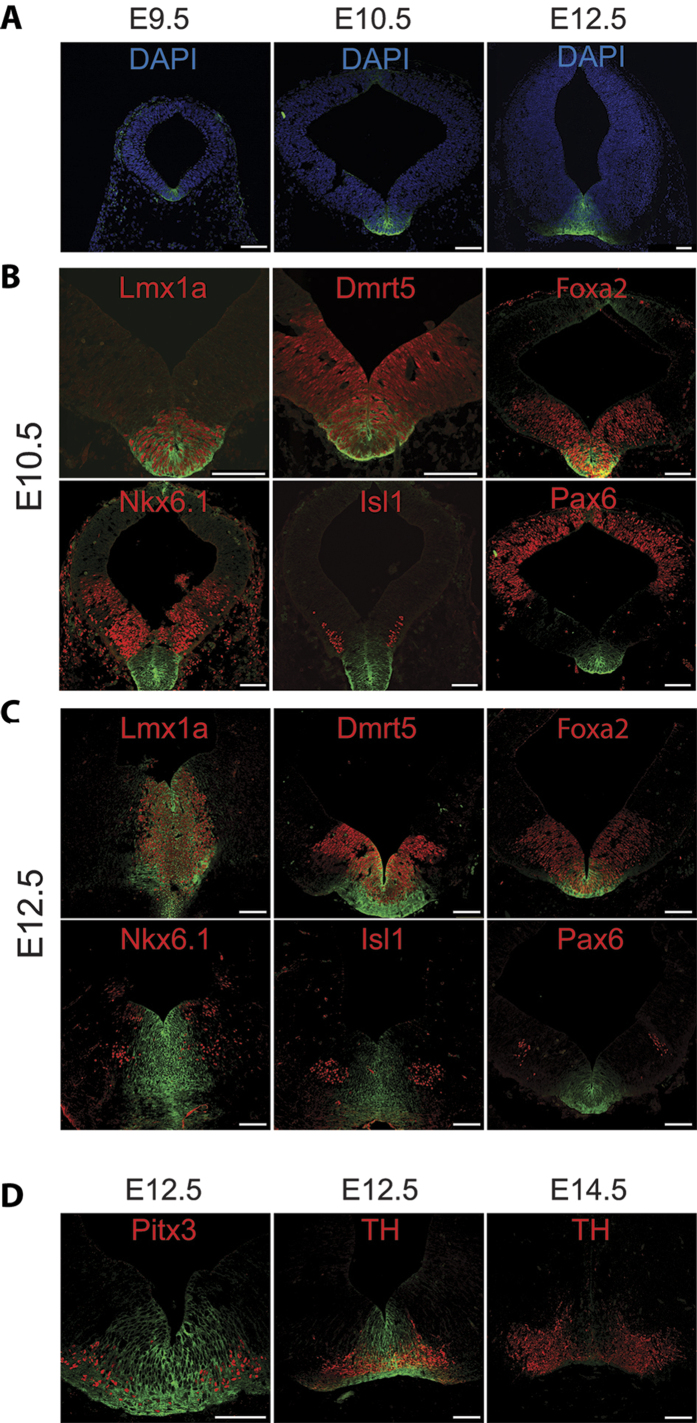
FolR1 expression in the midbrain. (**A**) Immunostaining of FolR1 in coronal sections of mouse ventral midbrains at E9.5, E10.5 and E12.5. FolR1 in green and Dapi nuclear counter stain in blue; (**B,C**) Double staining of FolR1 (green) with dopaminergic (Lmx1a, Dmrt5, Foxa2) and non-dopaminergic markers (Nkx6.1, Isl1, Pax6) in E10.5 and E12.5 mouse ventral midbrain sections, respectively; (**D**) Double staining of FolR1 (green) with Pitx3 and TH in E12.5 and E14.5 midbrain sections. All other markers are in red. Scale bars: 100 µm for all panels.

Immunofluorescence staining revealed highly restricted expression of FolR1in the mesencephalic floor plate, the brain region that give rise to mesDA neurons (Fig. 1A). FolR1 expression was firstly observed in E9.5, which is about one day later than that of Lmx1a, the expression of which is considered to encode the mesDA fate^18^. To ascertain that FolR1 marks cells of the mesDA lineage, we examined co-expression of FolR1with several dopaminergic and non-dopaminergic markers in the developing midbrain by double immunohistochemical staining (Fig. 1B–D). We found that the FolR1^+^ domain completely overlaps with the Lmx1a^+^ domain at both E10.5 and E12.5 (Fig. 1B,C). Neural progenitors expressing Dmrt5 and Foxa2 occupy a broader domain in the ventral midbrain and give rise to both mesDA and non-mesDA progeny^16^,^19^. We found that only the most medial part of the Foxa2^+^Dmrt5^+^ domain contained FolR1^+^ cells (Fig. 1B,C). In contrast, FolR1exhibited mutually exclusive or non-overlapping expression pattern to that of Nkx6.1, Islet1 and Pax6, which are transcription factors encoding non-dopaminergic neuroepithelial cells (Fig. 1B)^20^. No FolR1 staining was detected in other parts of the brain, except weak labelling in the dorsal midline cells at E10.5 (Fig. 1A).

To investigate whether FolR1 expression is maintained in differentiated postmitotic mesDA neurons, we performed double staining of FolR1 with Pitx3 and tyrosine hydroxylase (TH) at E12.5 and E14.5, the developing time window when mesDA neurogeneis is most active (Fig. 1D). Pitx3 is a transcription factor that co-expresses with TH in nascent postmitotic and mature mesDA neurons in the adult brain^21^. We found a partial overlap between FolR1^+^ and Pitx3^+^ and TH^+^ cells (Fig. 1D). The single Pitx3^+^ or TH^+^ cells were mostly located laterally to the midline. As development progress to E14.5, FolR1 expression was almost undetectable in TH^+^ neurons (Fig. 1D). Together, our data demonstrate FolR1 as a specific cell surface marker for mesDA neural progenitors and nascent mesDA neurons.

### FolR1 expression in PSC-derived mesDA lineage

**Figure 2:**
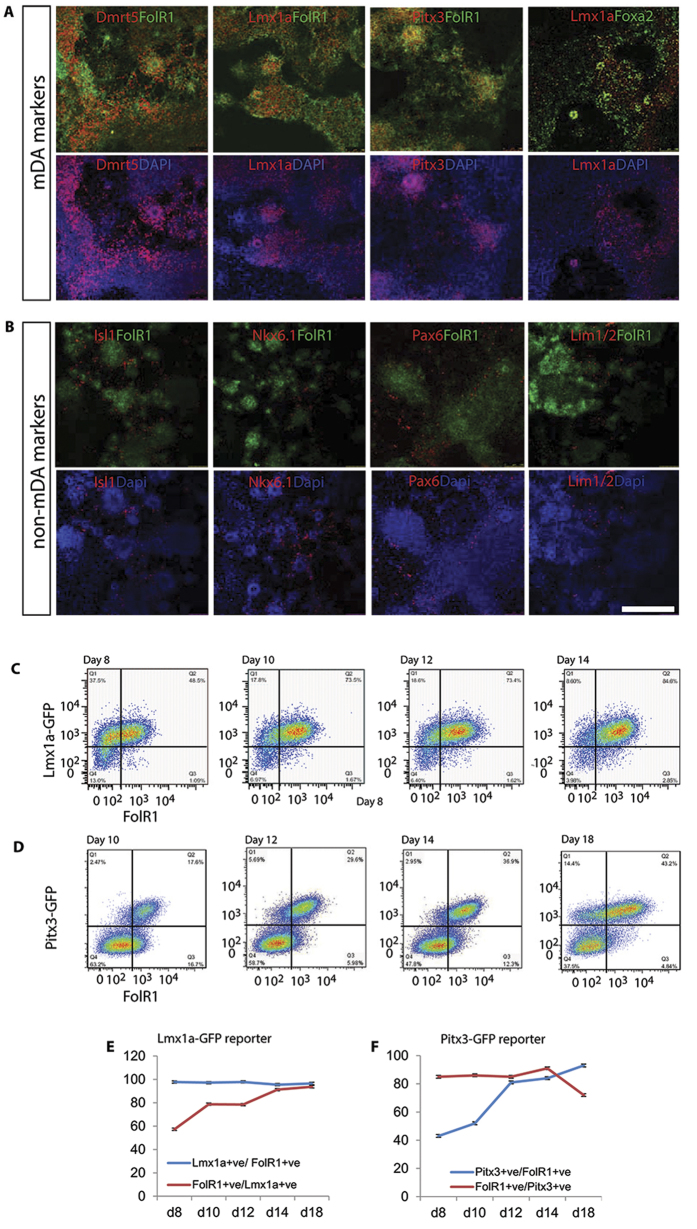
FolR1 expression in stem-cell derived mesDA neural progenitors. Cells were fixed after 10 days of mesDA differentiation and double stained for FolR1 (green) and other markers (red). (**A**) Widespread co-expression of FolR1 with mesDA lineage markers Dmrt5, Lmx1a and Pitx3; (**B**) Mutually exclusive expression of FolR1 and non-dopaminergic genes expressed in the ventral lateral midbrain Isl1, Nkx6.1, Pax6 and Lim1/2; (**C,D**) Flow cytometry analysis of FolR1 antibody stained mesDA cultures derived from Lmx1a-GFP and Pitx3-GFP reporter mESCs, respectively, at different time points; (**E,F**) Quantitative analysis of temporal expression profile of FolR1 represented in (**C,D**), respectively.

To investigate whether the developmental expression of FolR1 is mirrored in PSC-derived mesDA cells, we induced mouse ESC differentiation towards mesDA fate using a monolayer culture protocol developed previously^8^. This protocol generates highly enriched mesDA neural progenitors at around day 8–10, and postmitotic mesDA neurons at day 12–14. As an initial investigation, we performed double immunocytochemistry of day 10 cultures using antibodies against FolR1 in conjunction with mesDA and non-mesDA neural progenitor markers. Consistent with the findings in the mouse brain, the majority of ESC-derived FolR1^+^ cells co-labelled with Lmx1a, Dmrt5, and Pitx3 (Fig. 2A). In contrast and in keeping with findings in the developing midbrain brain, FolR1 did not co-stain with non-mesDA markers including Nkx6.1, Islet1, Pax6 or Lim1/2 (Fig. 2B). These data suggest that FolR1 also marks the mesDA lineage in ESC-derivatives.

To further map FolR1 temporal expression during the course of mESC mesDA neural differentiation, we analysed FolR1 protein expression in neural derivatives of two reporter mouse ESC lines that express GFP under the control of the endogenous *Lmx1a* or *Pitx3* promoter, respectively, by immunostaining of FolR1 followed by flow cytometry analysis. The use of these reporter cells allow the visualisation of ESC-derived mesDA neural progenitors (*Lmx1a*-GFP) and nascent postmitotic mesDA neurons (*Pitx3*-GFP) in real time during the course of *in vitro* differentiation via GFP expression^8^,^22^,^23^. When analysing the *Lmx1a*-GFP cultures, we found that the vast majority of the FolR1^+^ cells were *Lmx1a*-GFP^+^ between day 8–14 (Fig. 2C,E). In contrast, not all *Lmx1a*-GFP^+^ cells were FolR1^+^. However, we observed a progressive increase in the proportions of FolR1^+^ cells in the *Lmx1a*-GFP^+^ fraction (57.3% ± 0.8% at day 8 vs 91.2 ± 0.7% at day 14, Fig. 2C,E), as shown by the steady shift of cells in the *Lmx1a*-GFP single positive fraction to the *Lmx1a*-GFP^+^ FolR1^+^ double positive fraction (Fig. 2C). These data provide further support that FolR1^+^ cells descend from the Lmx1a^+^ mesDA progenitors.

Pitx3 expression in the brain is exclusive to mesDA neurons, with the onset of its expression proceeds slightly to that of TH during foetal development^21^. In comparison to that observed with the *Lmx1a*-GFP cultures, the Pitx3-GFP reporter gave a very different temporal expression pattern relative to FolR1 (Fig. 2D,F). We observed a steep increase in the number of *Pitx3*-GFP^+^ cells within the FolR1^+^ population (43% ± 4% at day 8 vs 93.7 ± 1% at day 18, Fig. 2D,F). This is in stark contrast to the constant and high content of *Lmx1a*-GFP^+^ cells in the FolR1^+^ population during the same differentiation time window (Fig. 2E). Moreover, the majority of *Pitx3*-GFP^+^ cell population were FolR1^+^ at day 8, and remained at this high level till day 14. At day 18 when the cultures were more mature, we observed a decrease in the number of FolR1^+^ cells within the Pitx3-GFP^+^ population. Together, these observations support the notion that FolR1^+^ cells give rise to Pitx3 expressing mesDA neurons and that the expression of FolR1 down regulates as mesDA neurons mature.

We then attempted to confirm FolR1 expression in human ESC-derived mesDA lineage cells by immunocytochemistry. We examined two developmental stages when FolR1 expression was found in the mouse cells. At day 20 of our hESC mesDA differentiation protocol^8^, the vast majority of the cells were mesDA neural progenitor as demonstrated by the combinatorial expression of LMX1A and DMRT5 (Fig. S2A). These progenitors gradually differentiate into postmitotic FOXA2^+^TH^+^ mesDA neurons from day 30 onwards (Fig. S2B). Unfortunately, none of the four FolR1 antibodies we have tested produced a specific staining pattern that matches that of the above mesDA lineage markers. However, RT-PCR analysis revealed that FolR1 transcript level is higher in hESCs differentiated following the mesDA protocol than that of cortical differentiation protocol both at day 20 and day 30^24^, suggesting that FolR1 expression is likely conserved in human mesDA cells.

### FolR1-based FACS isolation of dopaminergic cells

**Figure 3:**
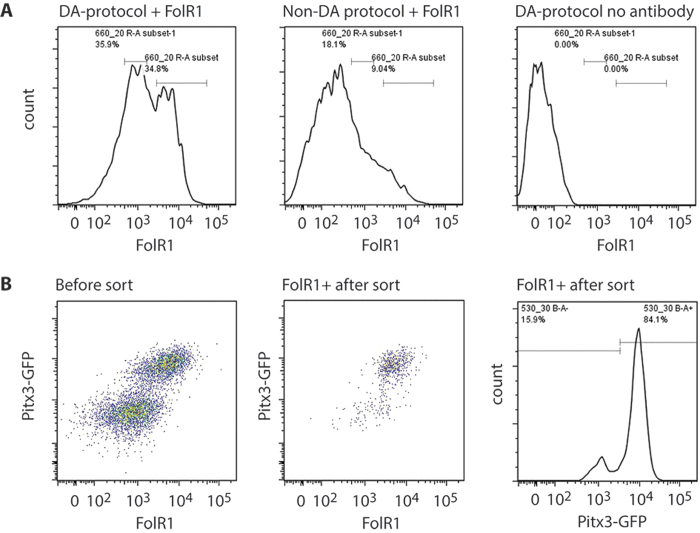
FACS purification of FolR1 labelled cells. (**A**) A representative flow cytometry profile for FolR1-labelled cells generated according to a dopaminergic (left) or non-dopaminergic differentiation protocol (middle), and an unstained control of cells differentiated according to a dopaminergic protocol (right); (**B**) Flow cytometry analysis of FolR1 antibody-labelled Pitx3-GFP reporter cells differentiated according to a dopminergic protocol. Left: scatter plot of the cells prior to FACS sorting, Middle: scatter plot of the cells straight after sorting, Right: a histogramme showing Pitx3-GFP expression profile by FACS sorted FolR1^+^ cells. All analysis were performed at day 13 of differentiation.

We next evaluate the feasibility of using FolR1 antibody as a tool to isolate ESC derived mesDA population by FACS. We chose day 13 cultures as the experimental material because both FolR1^+^ mesDA progenitors and nascent post-mitotic mesDA neurons are present at this stage. For the convenience of quality control analysis, we performed the initial experiment using cultures derived from the Pitx3-GFP reporter mESCs. ESCs were differentiated using the mesDA protocol as well as well as the (non-dopaminergic) standard monolayer differentiation condition without any exogenous inductive molecules. FolR1 antibody stained cells from the control differentiation and the mesDA differentiated culture without FolR1 antibody staining were used as negative controls for setting up the sorting gate (Fig. 3A). Post-sort flow cytometry analysis confirmed that the majority (>80%) of the sorted cells express a high level of FolR1 with the remaining cells lower FolR1-expressing cells (Fig. 3B). Furthermore, FACS sorted FolR1^+^ cell population was highly enriched for Pitx3-GFP expressing cells (Fig. 3B).

**Figure 4:**
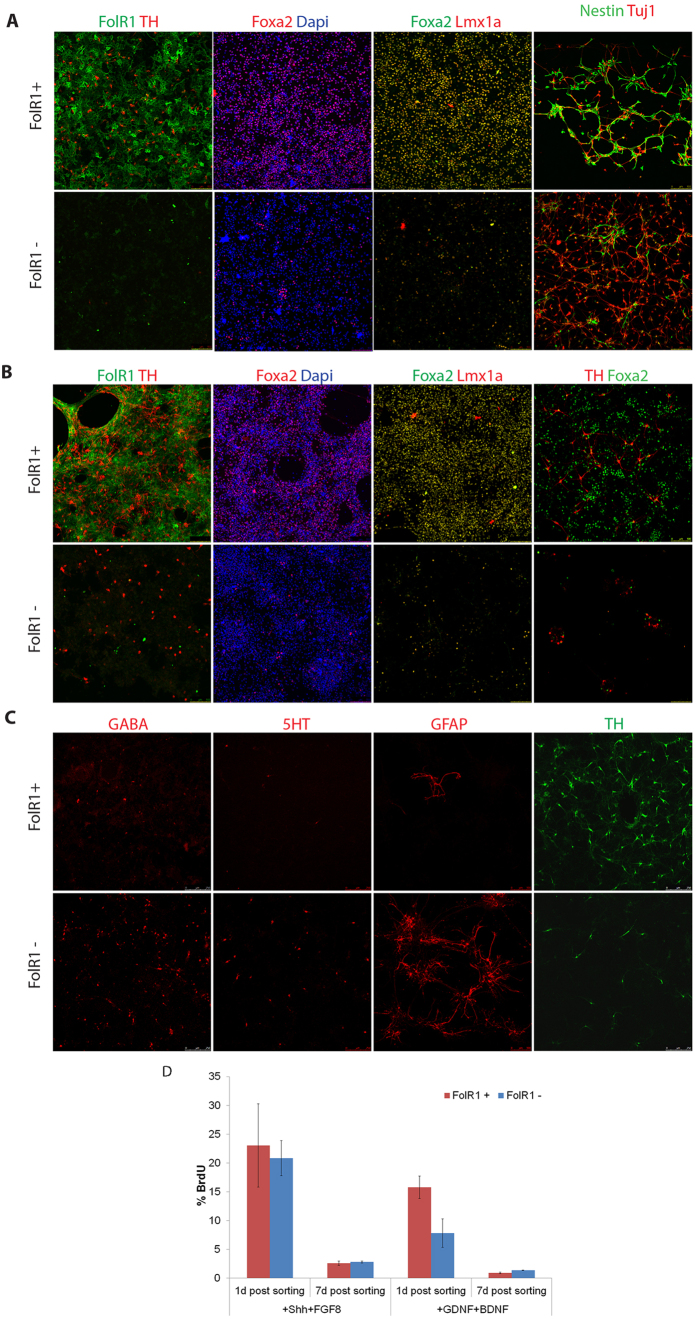
Characterisation of FolR1 sorted cells. Cells were sorted on day 13 and cultured for 1 and 7 days after sorting. (**A**) 1 day cultured cells stained for ventral midbrain markers; (**B**) 7 day cultured cells stained for ventral midbrain markers; (**C**) 7 day cultured cells stained for GABA (GABAeric), 5HT (serotonergic) neuron and GFAP (astrocytes); (**D**) BrdU incorporation of sorted FolR1^+^ cells. Cells were sorted on day 10 of *in vitro* differentiation and subsequently cultured either in maturation media (+GDNF, BDNF) or in proliferation-promoting media (+SHH, +FGF8). BrdU was added for 3 hours before fixation at day 1 and day 7 post-sort, respectively.

To gain further support that FolR1 based FACS purification enrich mesDA neurons in their derivatives, we sorted FolR1^+^ and FolR1^−^ cells from wild type E14TG2a mESC derivatives and replated the sorted cells in neuronal differentiation media and cultured for 1 or 7 days, respectively. Double immunostaining revealed that, at one day post sort, TH^+^ mesDA neurons were already detectable in the FolR1^+^ fraction but the major population were neural progenitors as demonstrated by the abundance of nestin^+^ cells and continued FolR1 expression (Fig. 4A). The vast majority of the cells expressed Foxa2 and Lmx1a, confirming the mesDA identity (Fig. 4A). In contrast, few FolR1^+^, Foxa2^+^, Lmx1a^+^ cells were detected in the FolR1^−^ counterpart. These cultures were dominated by Tuj1^+^ young neurons with a minority of Nestin^+^ cells. However, no TH^+^ cell were found in the FolR1^−^ fraction one day post sorting. At 7 days post sorting, evident enrichment in Foxa2^+^Lmx1a^+^ cells in the FolR1^+^ fraction remain the same to that of day 1 post-sort cultures (Fig. 4B). However, there was evident increase in the number of TH^+^ cells and the intensity of FolR1 staining was weaker than day 1 (Fig. 4B). In contrast, the FolR1^−^ fraction had fewer TH^+^ neurons, and Foxa2^+^ or Lmx1a^+^ cells were rare (Fig. 4B). To further evaluate the cellular identity of the FolR1^−^ fraction, we stained day 7 post-sort cultures with antibodies against GABA (GABAergic neurons), 5-hydroxytryptamine receptors (5-HT, serotonergic neurons) and glial fibrilary acidic proteins (GFAP, astrocytes). In stark contrast to enriched TH^+^ cells in the FloR1^+^ fraction, cells positive to these markers were preferentially found in the FolR1^−^ fraction (Fig. 4C).

The increased numbers of TH^+^ cells in day 7 post-sort versus day 1 post-sort is reversely correlated with a down regulation of FolR1 in these cells. We therefore investigated the proliferative capacity of FACS-sorted cells by BrdU labelling (Fig. 4D). Cells were cultured either in the presence of Sonic hedgehog (Shh) and FGF8, a condition that support the maintenance of mesDA progenitors, or in the presence of GDNF and BDNF, a condition that promote terminal differentiation. We found that over 20% (21 ± 3%) of the FolR1^+^ cells incorporated BrdU at day 1 whereas less than 3% (2.8 ± 0.2%) were BrdU labelled at day 7. In contrast, fewer cells incorporated BrdU when cultured in differentiation condition at both day 1 (7.8 ± 2.5%) and day 7 (1.4 ± 0.03%). These data are consistent with the temporal expression profile of FolR1 in mesDA progenitors of the ventral midbrain.

### Enriching dopaminergic cells by magnetic cell sorting (MACs)

**Figure 5:**
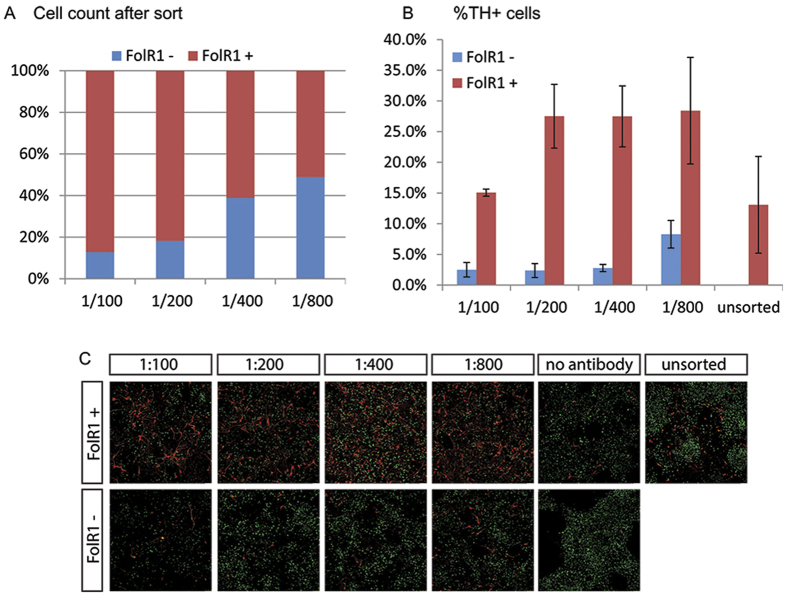
Magnetic cell sorting FolR1 antibody. (**A**) MACs sorting efficiency of day 13 culture using different antibody dilutions; (**B**) Quantitative analysis for TH^+^ cells in day 7 post-sort cultures derived from the FolR1^+^ and FolR1^−^ fractions; (**C**) Immunostaining for TH (red) and NeuN (green) in FolR1^+^ and FolR1^−^ sorted cells at different antibody concentrations.

Although FACS is a frequently used method for cell purification and provides more possibilities for choice of colours (markers), it does require accessibility and some coordination with the facility. We therefore tested a simpler method that could be done by everyone on a bench: magnetic cell sorting (MACs). MACs is also more gentle on the cells in comparison to FACS. Because magnetic microbeads conjugated with a FolR1 antibody has yet to be developed as a commercial product, we employed an indirect labelling approach involving the use of anti-biotin labelled magnetic microbeads and homemade biotinylated FolR1 antibody. Four dilutions of biotinylated FolR1 antibody were evaluated for MACs on day13 cultures and the beads-bound and unbound fractions of cells were counted after MACs. We found that the proportion of beads-bound cells increased in line with increasing concentrations of the FolR1 antibody, with 86%, 80% and 60% of the total cells fractioned as FolR1^+^ population at 1:100. 1:200 and 1:400 dilutions, respectively (Fig. 5A).

To investigate the fidelity of the MACs sort, post-sort cells were cultured for 7 days and were then immunostained for the expression of TH (Fig. 4B,C). We observed an enrichment of TH^+^ neurons in the beads-bound fraction under all MACs conditions compared to mock sorted (no antibody) or unsorted controls. The condition with 1/200 antibody dilution yielded the highest ratio of TH^+^ neurons between the beads-bound and unbound cell fractions and has the lowest (<3%) numbers of ‘contaminating’ TH^+^ neurons in the unbound cell population (Fig. 5B,C). However, the numbers of TH^+^ neurons in the unbound fraction increased by three fold when the antibody was diluted at 1/800, compared to other three dilutions tested (Fig. 5B,C). Further analysis of MACs sorted cells under 1/200 condition confirmed enrichment of Foxa2^+^Lmx1a^+^ neural progenitors at one day post MACs (Fig. S3A). Moreover, non-dopaminergic neurons were preferentially found in the unbound cell cultures analysed 7 days post MACs, as with the FACS sorted cells (Fig. S3B). These data provided clear indication that magnetic sorting with the FolR1 antibody is able to enrich cells of the mesDA lineage. Together, the FACS and MACs data demonstrate that FolR1 can be used as a valuable marker for the purification of live mesDA progenitors.

## Discussion

Isolation of defined cell populations using cell surface markers is a well-established practice for several decades. One good example is the purification of haematopoietic stem cells for bone marrow transplantation for treating leukaemia. Parkinson’s disease is one of the top targets for transplantation based cell therapy and disease modelling with patient iPSCs. Significant progress has been made recently in developing efficient methods for generating ‘authentic’ mesDA neurons from hPSCs which showed promising *in vivo* survival and function following transplantation in animal models of Parkinson’s diseases in prof of principle studies^1^,^2^,^8^. However, risk in tumour growth from residual PSCs or intermediate progenitors, adverse effects and/or compromised efficacy due to the presence of phenotypically undefined cells in PSC-derivatives remain a realistic possibility. Using purified donor cells would provide the necessary safeguard against these shortcomings^25^.

Many lines of thorough pre-clinical investigations demonstrated that post-graft survival and the efficacy of functional repair depends critically on the developmental status of the donor cells^9^,^26^. Foetal derived dopaminergic fated mitotic progenitors were shown to survive and integrate better than differentiated postmitotic mesDA neurons following transplantation into the host brain^9^,^13^,^27^. The onset of FolR1 expression mirrors closely to that of Lmx1a in the ventral midbrain and coincides with mesDA fate specification and neurogenesis. Hence FolR1-labelled cells are committed mesDA precursors and nascent mesDA neurons. This temporal expression profile also appears to be similar to that of Corin and FP4^10^. Corin^+^ cells isolated either from rodent foetal mesencephalic tissue or differentiated ESCs have been shown to survive and differentiate into TH^+^ neurons in animal models of Parkinson’s disease^13^,^14^,^15^. Together, these findings suggest that FolR1^+^ mesDA neural progenitors are within the developmental time window suitable for transplantation. Because the expression of Corin and FP4 extend to the floor plate of the hindbrain and spinal cord, FolR1 offers better spatial specificity.

A recent study reported four new mesDA markers: Alcam, Chl, Igsf8 and Gfra^17^, the first three of which were also identified in the current study (Table S1). The expression of these markers appears to come on later than FolR1 and are not restricted to the mesDA cell population. Bye *et al*. showed that Alcam^+^ and Chl^+^ cells purified from E15 rat ventral midbrain can survive and mature into TH expressing neurons in 6-OHDA lesioned rat model of Parkinson’s disease. It would be interesting in future studies to directly compare the transplantation efficacy of cells isolated based on FolR1, Alcam and Chl, given the differences in differentiation status these molecues mark.

It has been shown that different iPSCs lines, either from the same or different individuals, can vary in their differentiation propensity^3^. Such system noise may mask cellular phenotype or lead to false pathology. To reduce system variation, cell purification has been employed in patient iPSC disease modelling of Parkinson’s disease^28^. In the case of Woodard *et al*., neural progenitors and young neurons isolated using CD133 (prominin-1) and CD56 (NACAM) were the subject of phenotypic analysis. FolR1 based sorting would allow specific comparison of mesDA lineage, as opposed to pan neural cells as reported^28^. Preliminary analysis suggest that FolR1 RNA is preferentially expressed in hESC-derived mesDA cells, although we haven’t been able to validate this finding at protein level in single cells due to a lack of FolR1 antibody that recognise human cells.

## Materials and Methods

### Mouse ESC culture and neural differentiation

Three mouse ESC lines were used: E14tg2a, Pitx3-GFP^21^ and Lmx1a-GFP^8^,^18^. Mouse ESCs were maintained in 0.1% gelatin coated flasks with GMEM supplemented with 10% FCS, NEAA, L-Glutamine, Sodium Pyruvate, 2-mercaptoethanol and LIF and passaged every other day. For monolayer neural differentiation, cells were plated at low density (7,500 cells cm^2^) in 50:50 ratio of DM/F12 supplemented with N2 and neurobasal supplemented with retinol-free B27 (referred to as N2B27 media). Cultures were then exposed to MEK inhibitor PD0325901 (1 μM, Axon) from day 2–4 and followed by Shh (200 ng/ml, C25 II-N, R&D) and FGF8b (100 ng/ml, Peprotech) from day 6–10. Media was changed every other day and at day 6 of differentiation, cells were split 1:3, replated into Poly-D-lysine and laminin-coated dishes, and maintained in N2B27 media^8^.

### hESCs culture and neural differentiation

H7 hESCs were maintained in BD Matrigel-coated plates in mTesR1 medium (StemCell Technologies) and passaged every 3–4 days. Monolayer neuronal differentiation was performed to induce cortical glutamatergic and midbrain dopaminergic neurons, respectively, according to published literatures^8^,^29^. Briefly, hESCs were subjected to dual SMAD inhibition with SB431542 (10 uM, Tocris) from day 0 to day 3, LDN193189 (100 nM, Tocris) and Dorsomorphin (200 nM, Tocris) from day 0 to day10. For cortical differentiation, cultures were then maintained in N2B27 media until the end of differentiation without additional patterning molecules. For generating dopamine neurons, cells were exposed to the MEK inhibitor PD0325901 (1 mM, Axon) between days 3–7, followed by Shh (200 ng/ml, C25 II-N, R&D), Purmorphamine (1 μM, Tocris) and FGF8b (100 ng/ml, Peprotech) from day 7 to day 11. Postmitotic dopaminergic neurons were maintained in N2B27 media supplemented with BDNF (10 ng/ml Peprotech), GDNF (10 ng/ml, Peprotech) and AA (0.2 mM, Sigma). At days 10 and 20, cells were passaged on Fibronectin and Poly-d-lysine/laminin-coated dishes, respectively.

### Immunocytochemistry

Cells were rinsed in PBS and fixed in 4%PFA for 15 mins. Cells were then permeabilised via three washes in PBS containing 0.3% Triton-X-100 (PBST) and then blocked in PBST containing 1% BSA and 3% normal donkey serum. Primary antibodies were added in blocking solution for 2 hours at ambient temperature or overnight at 4 °C. The cells were washed in PBST three times before being incubated for 1 hour in the dark in Alexa-Fluor secondary antibodies, 1:200 (Invitrogen). Three PBST washes were then performed that included one with DAPI, 1:1000 (Molecular Probes). The primary antibodies used in this study were: FolR1(sheep, R&D), TH (rabbit, PelFreez), Pitx3 (rabbit, gift of M. Smidt, University of Amsterdam), Foxa2 (goat, Santa Cruz), Foxa2 (Rabbit, Abcam), Lmx1a (rabbit, gift of M. German, UCSF), Nestin (mouse, BD Pharmagen), GFAP (rabbit, Dako), GABA(rabbit, Sigma), 5HT(mouse, Abcam), Isl1(mouse, DSHB), Lim1/2(mouse, DSHB), Pax6(mouse, DSHB), Nkx6.1(mouse, DSHB), Dmrt5(rabbit, custom made). Images were taken on a Leica TCS SP5 confocal microscope. Quantification of markers was carried out manually by examining randomly selected fields from at least 3 independent experiments and presented as means ± sem.

### Fluorescence-activated cell sorting (FACS)

Cells were dissociated with accutase into a single cell suspension in DPBS. After one wash by centrifugation, the cell pellet was resuspended in blocking solution at 10^7^ cells/ml and incubated for 20 minutes at RT. Cells were then labelled with FolR1 antibody conjugated with the CF640R dye using the ‘Mix-n-Stain CF640R’ kit as per the supplier’s instructions (Biotium). Cells were washed twice with DPBS and resuspended in 1 ml of sorting buffer containing 1 mM EDTA, 25 mM HEPES pH 7.0 and 1% FCS in DPBS. The stained cells were analysed or sorted on a FACSAria (Becton Bickinson Biosciences) using a FACS Diva software v6.1.3. The gate for FolR1-negative cells was set using a no antibody control of the same cell preparation as well as FolR1 stained cells derived from a control (non-dopaminergic) differentiation protocol.

### Magnetic cell sorting (MAC)

Dissociated cells were labelled as described above but with biotinylated FolR1 antibody prepared using the ‘One-step antibody Biotinylation kit’ (Miltenyi Biotec, 130-093-385). The labelled cells were then incubated with Anti-Biotin Microbeads for 15 minutes on ice. Cells were then washed once in sorting buffer and resuspended in 500 µl sorting buffer. FolR1^+^ cells were separated from the FolR1^−^ cells using the MS columns on a MiniMACS Separator (Miltenyi Biotec) following the Manufacture’s instructions.

### Quantitative (q) PCR

Total RNA was extracted using TRI Reagent (Sigma) following the manufacturer’s protocol. cDNA was synthesised using the SuperScript^®^ III Reverse Transcriptase (Thermo Fisher) and purified from genomic DNA with the Turbo DNA-free kit (Ambion). RT-qPCR was performed on a Biorad CFX96 detection system in a 20 μl reaction, containing 1x MESA GREEN (Eurogentech) and 500 nM primers (Sigma). All PCR data were normalized to the reference gene GAPDH. For PCR primers, see Table S2. All qPCR data are presented as mean ± SD.

## Additional Information

**How to cite this article**: Gennet, N. *et al*. FolR1: a novel cell surface marker for isolating midbrain dopamine neural progenitors and nascent dopamine neurons. *Sci. Rep.* **6**, 32488; doi: 10.1038/srep32488 (2016).

## Supplementary Material

Supplementary Information
