# Supplementary material for: FolR1: a novel cell surface marker for isolating midbrain dopamine neural progenitors and nascent dopamine neurons
Source: Sci Rep. 2016 Sep 1;6:32488. doi: 10.1038/srep32488 (PMC5007511; doi:10.1038/srep32488)
Supplement: Supplementary Information [file srep32488-s1.pdf]

FolR1: a novel cell surface marker for isolating midbrain dopamine neural progenitors and nascent dopamine neurons

Nicole Gennet, Claudia Tamburini, Xinsheng Nan and Meng Li[#]

Neuroscience and Mental Health Research Institute, School of Medicine and school of Bioscience, Cardiff University, Cardiff, CF24, 4HQ, UK

[#]Corresponding Author: lim26@cf.ac.uk.

Running title: Purification of dopamine progenitors by FolR1

keywords: midbrain dopamine neurons, stem cells, surface marker, enrichment, cell sorting

Supplemental information

FolR1: a novel cell surface marker for isolating committed midbrain dopamine neural progenitors and nascent dopamine neurons

Gennet et al.,

FigS1

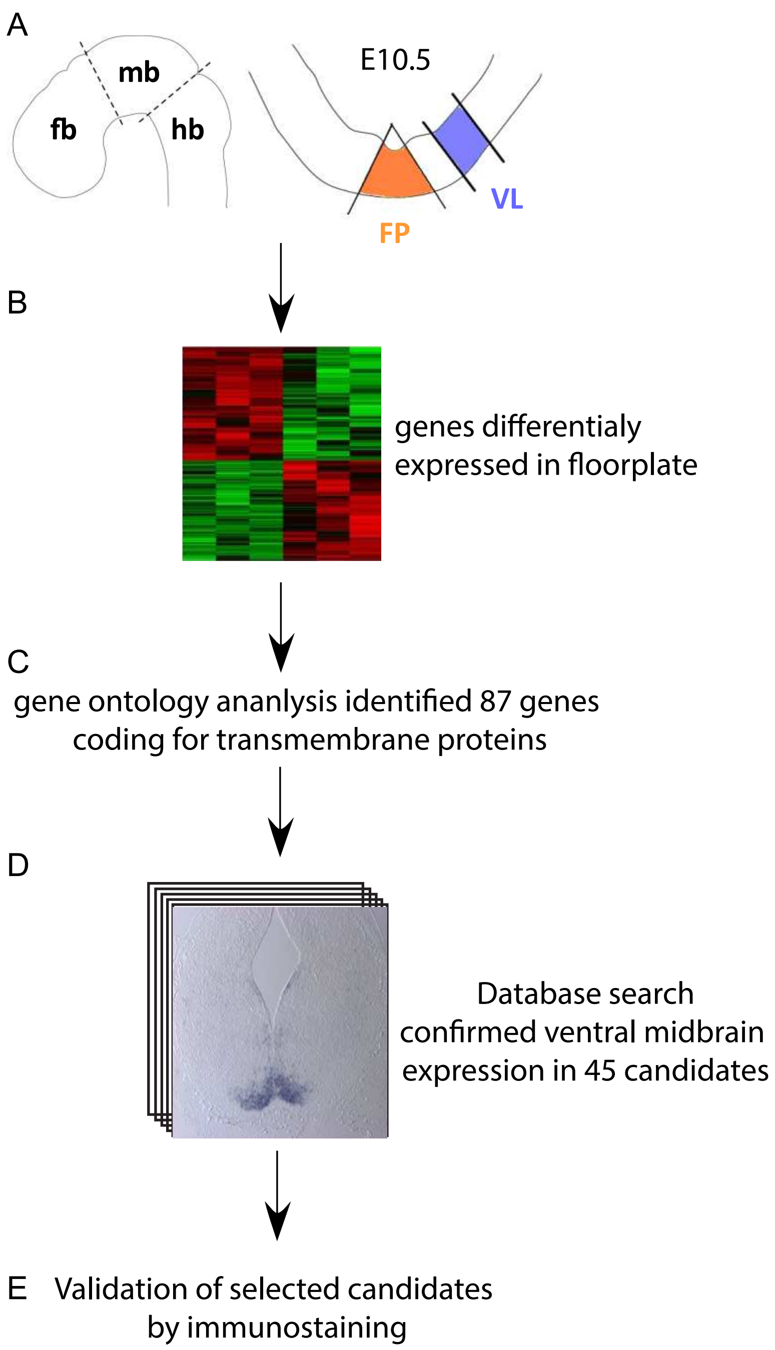

Gennet et al., Fig S2

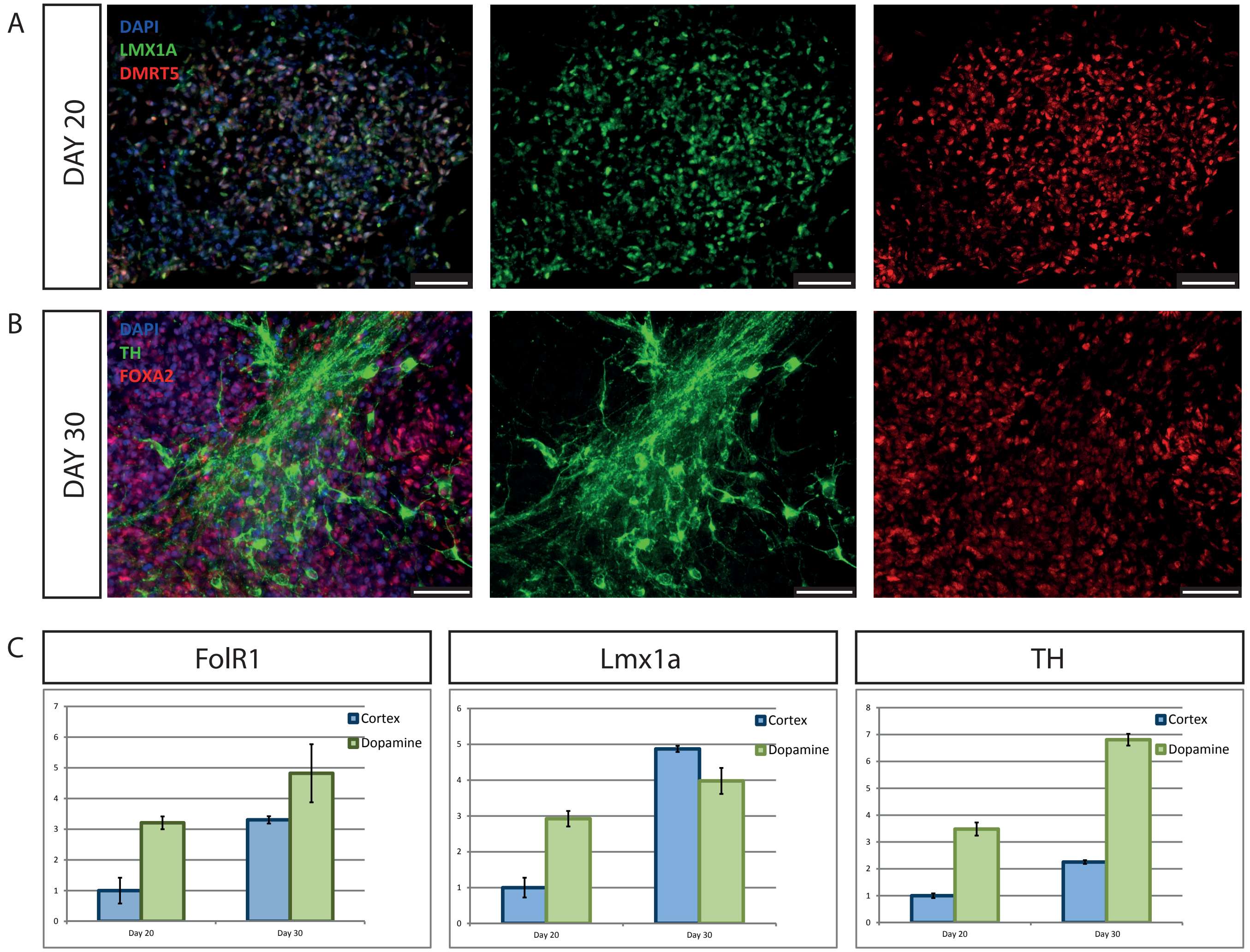

Gennet et al., FigS3

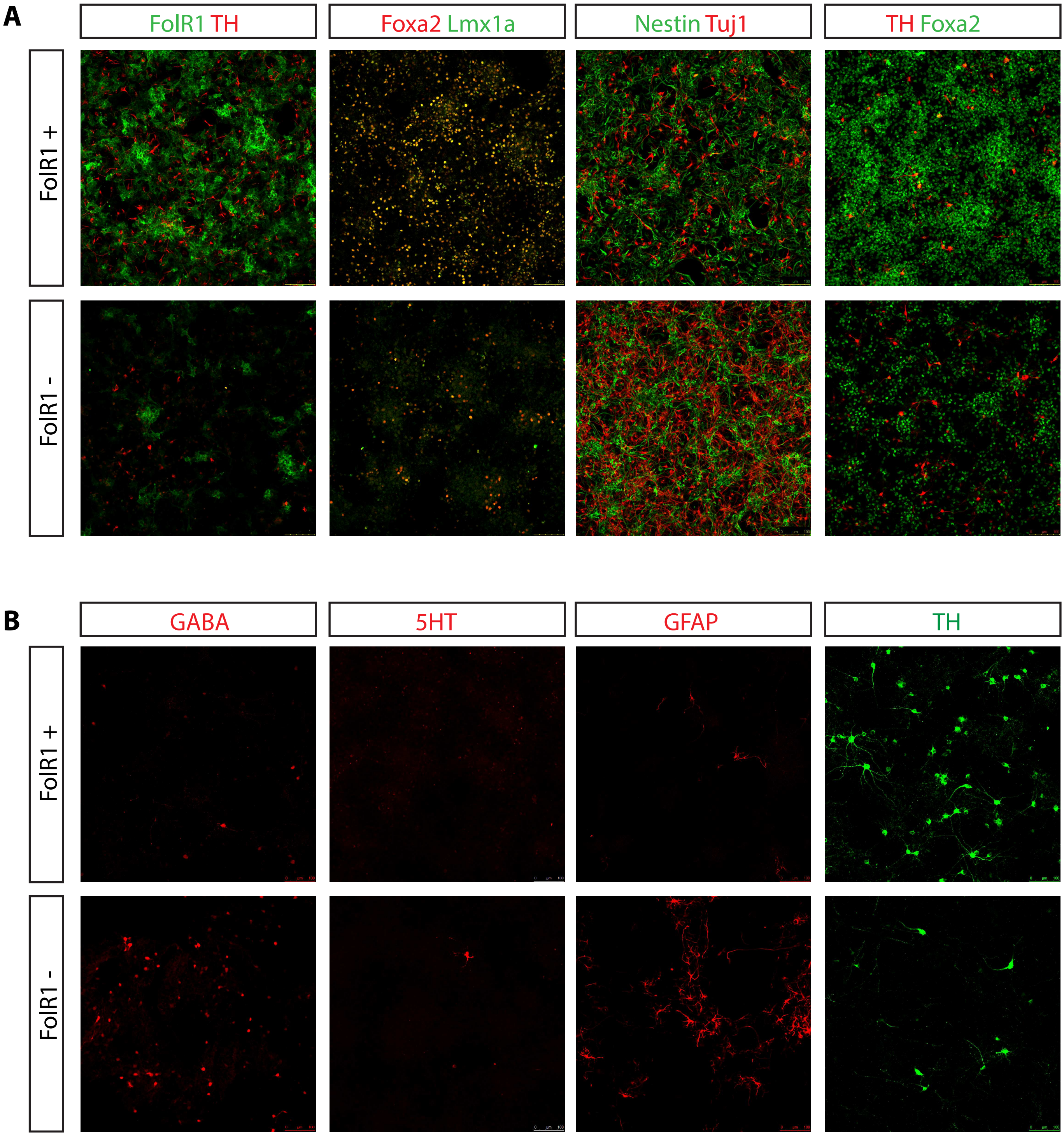

Table S1, Membrane coding genes preferentially expressed in the floor plate of mouse E10.5 midbrain

Probe Set ID	Entrez Gene	Gene Symbol
1419393_at	27409	Abcg5
1425102_a_at	70008	Ace2
1425103_at	70008	Ace2
1450658_at	100048332 /// 23794	Adamts5 /// LOC100048332
1429022_at	11517	Adcyap1r1
1426300_at	11658	**Alcam**
1448213_at	16952	**Anxa1**
1419091_a_at	12306	**Anxa2**
1443823_s_at	98660	Atp1a2
1440397_at	12293	**Cacna2d1**
1451499_at	320405	Cadps2
1418094_s_at	12351	**Car4**
1423286_at	12404	Cbln1
1421195_at	12425	Cckar
1423760_at	12505	**Cd44**
1450288_at	12563	**Cdh6**
1435190_at	12661	**Chl1**
1457008_at	108015	**Chrnb4**
1441165_s_at	64085	**Clstn2**
1429210_at	237759	Col23a1
1424131_at	12835	Col6a3
1419017_at	53419	**Corin**
1429413_at	70574	**Cpm**
1453009_at	65969	Cubn
1426990_at	13405	**Dmd**
1452270_s_at	13599	**Ecel1**
1417307_at	13617	Ednra
1448665_at	13649	**Egfr**
1422586_at	140580	**Elmo1**
1433525_at	18606	**Enpp2**
1424932_at	18606	F2rl1
1446610_at	14063	Fcgrt
1415894_at	14132	**Folr1**
1448136_at	14275	**Folr2**
1448931_at	14276	**Ghr**
1416978_at	14600	Gpr120
1450995_at	14600	**Gpr37**
1451648_a_at	107221	**Gprc5c**
1417962_s_at	14763	**Hcrtr1 /// LOC100046129 /// LOC100048817**
1460675_at	140559	**Igsf8**

1451501_a_at	70355	**Il1rap**
1450875_at	16180	Irs1
1452947_at	16180	Itga3
1421844_at	16367	Itgb5
1421997_s_at	16419	Kcnj10
1455158_at	16419	Kcnq5
1417534_at	16513	**Kctd4**
1457587_at	17311	**Kitl**
1420537_at	17311	Lama2
1441801_at	17311	Lifr
1426152_a_at	16880	LOC100047693 /// Pvrl3
1426285_at	100047693 /// 58998	**Lpl**
1454984_at	100047693 /// 58998	Lsr
1421132_at	16956	Myo16
1421133_at	16956	**Ncam2**
1423331_a_at	54135	Negr1
1415904_at	244281	**Notch2**
1451255_at	320840	**Nrcam**
1445532_at	18129	**Nrp1**
1455556_at	18186	Nrp2
1434709_at	18186	Odz3
1418084_at	18187	P2rx6
1448943_at	18187	Pcdh8
1448944_at	23965	Pdk1
1429178_at	228026	Pkp2
1456925_at	228026	**Plxdc2**
1435836_at	67448	**Pvrl3**
1449799_s_at	67448	**Pygl**
1418912_at	58998	Rab15
1449270_at	110095	**Ralgps2**
1448673_at	78255	**Rgnef**
1417230_at	110596	Scn11a
1428789_at	110596	Sgms1
1419457_at	24046	Sirpa
1419458_at	208449	**Slc1a3**
1420784_at	19261	**Slc24a3**
1436499_at	20512	Slc4a4
1436869_at	20512	Slc6a15
1416985_at	94249	**Steap2**
1424308_at	74051	**Sv2b**
1426712_at	74051	Synpo
1428636_at	64176	Syt12
1444290_at	104027	Thbd
1434800_at	171180	**Tm4sf1**

1435687_at	21809	**Tmem47**
1422878_at	17112	**Vldlr**

Table S2. PCR primers sequence

GENE	FORWARD	REVERSE
GAPDH	5-atgacatcaagaaggtggtg'-3'	5'-cataccaggaaatgagcttg-3'
FolR1	5- gaggctcagacaaggattgc-3'	5'-gtgggtgtggggaagtagaa-3'
Lmx1a	5'-gagaccacctgcttctaccg-3'	5'-gcccgcataacaaactcatt-3'
TH	5'-gagtacaccgccgaggagattg-3'	5-gcggatatactgggtgcactgg-3'

Legends for supplemental figures and table.

Fig S1 Gene expression profiling for ventral midbrain surface markers
A, Isolation of floor plate and ventrolateral tissue from E10.5 mouse midbrain; B, RNA microarray analysis for identifying genes preferentially expressed in the floorplate; C, identify genes coding for plasma membrane protein according to gene ontology database, D, Image database search to validate midbrain expression; E, Selection of the genes with best expression patterns and commercially available antibodies for immunohistochemical validation on E10.5 mouse midbrain sections.

Fig S2 Expression of FolR1 in hESC-derived mesDA cells
A, Day 20 culture double stained for mesDA neural progenitor marker LMX1A and DMRT5; B, day 30 culture stained for TH and FOXA2; C, RT-PCR analysis of day 20 and day 30 hESC differentiation cultures following mesDA and cortical differentiation protocols, respectively.

Fig S3 Immunostaining of FolR1+ and FolR1- cells after magnetic sorting
Day 13 cultures were processed for MACs using 1/200 antibody dilution and were cultured for a further 1 and 7 days, respectively. A, 1 day post-sort cultures stained for midbrain markers; B, 7 day post-sort cultures immunostained for markers for Astrocytes (GFAP) and markers for other neuronal neurotransmitter type (GABA, 5HT and TH).

Table S1: Membrane proteins preferentially expressed in E10.5 floor plate
Gene ontology analysis generated a list of membrane coding genes preferentially expressed in the floor plate of E10.5 mouse midbrain brains. Raw data obtained by microarray using the Affymetrix.GeneChip.Mouse430-2. The genes which midbrain expression is confirmed by image data search are labelled in bold.
